# Quantifying the Autonomy of Structurally Diverse Automata: A Comparison of Candidate Measures

**DOI:** 10.3390/e23111415

**Published:** 2021-10-28

**Authors:** Larissa Albantakis

**Affiliations:** Department of Psychiatry, University of Wisconsin–Madison, Madison, WI 53719, USA; albantakis@wisc.edu

**Keywords:** agency, artificial evolution, causation, integrated information, intelligence

## Abstract

Should the internal structure of a system matter when it comes to autonomy? While there is still no consensus on a rigorous, quantifiable definition of autonomy, multiple candidate measures and related quantities have been proposed across various disciplines, including graph-theory, information-theory, and complex system science. Here, I review and compare a range of measures related to autonomy and intelligent behavior. To that end, I analyzed the structural, information-theoretical, causal, and dynamical properties of simple artificial agents evolved to solve a spatial navigation task, with or without a need for associative memory. By contrast to standard artificial neural networks with fixed architectures and node functions, here, independent evolution simulations produced successful agents with diverse neural architectures and functions. This makes it possible to distinguish quantities that characterize task demands and input-output behavior, from those that capture intrinsic differences between substrates, which may help to determine more stringent requisites for autonomous behavior and the means to measure it.

## 1. Introduction

Agents are open systems that dynamically and informationally interact with their environment. In biological, evolved systems, more intelligent behavior is typically associated with greater autonomy from the environment. Simple systems are thought to act in an automated, reflexive manner, while intelligent organisms perform complex tasks in an autonomous, context-dependent way, and increasingly rely on internal states, such as memory, or learned, adjustable preferences. To date, however, a rigorous, quantifiable definition of “autonomy” and “autonomous actions” remains elusive [1,2,3,4,5].

What is more, our preconceived biological notions are being challenged by recent advances in artificial intelligence. As functional equivalence between biological brains and computers seems within reach, striking differences remain regarding their respective problem-solving algorithms, implementation, and causal structures. In particular, the classical feed-forward architecture of the most common artificial neural networks (ANNs) suggests that they are just “machines running through the motions”—not one unified entity [3,6,7,8,9,10], but a unidirectional chain of events. Yet, they achieve super-human levels of performance even in tasks that supposedly require creativity and intuition [11]. Does implementation matter when it comes to autonomy?

Here I will address this question by comparing the structural, informational, dynamical, and causal properties of evolved ANNs based on a range of state-of-the-art measures that have been proposed as quantities related to intelligence and autonomy, which I will review below. These quantitative measures specifically capture three aspects of autonomy: self-determination (how much the system determines its own internal states), closure (whether the system forms an independent entity from the environment), and agency (whether a system’s actions are determined by its internal mechanisms, as opposed to external influences), though other relevant aspects and classifications have been proposed in the literature [1,2,5,12,13,14] (see Discussion Section 5).

The particular ANNs used in this study are simple artificial agents evolved to solve a spatial navigation task, with or without a need for associative memory [15]. These agents are equipped with small, discrete neural networks (“Markov Brains”), whose connectivity and node functions adapted over the course of their evolution [16]. As a result, independent evolution simulations may produce agents with diverse neural architectures that all successfully navigate their environment, including feed-forward and recurrent ANNs. From the outside, the agents’ behavior should thus seem equally “intelligent”. However, the computational substrates that produced their behavior differ qualitatively in their neural mechanisms and connectivity.

Based on such a data set of structurally diverse automata, it becomes possible to distinguish quantities that primarily characterize task demands and input-output behavior (“what the agent is doing”), from quantities that capture differences between the various substrates (“how the agent is doing it”), beyond their adaptive performance. Such a distinction may help to determine more stringent requisites for what counts as autonomous behavior and the means by which it is measured. In particular, the idea that an autonomous system must form a unified whole that is to some degree independent of its environment (yet interacts with it) is widely acknowledged [3,6,7,8,9,10,17,18,19]. However, structural, informational, causal, or dynamical measures of “closure” from the environment do not necessarily go hand in hand. For example, whether a measure is evaluated based on observed activity (that is, information-theoretically) or system perturbations (for a causal analysis) may result in significantly different assessments of the degree to which an agent’s internal states determine its behavior (see also [1]). Which notion of “closure” is the relevant one for assessing autonomy? In addition, the role of internal states and memory for autonomy and intelligent behavior still remains unclear. Should memory of environmental states count towards higher levels of autonomy or should it be discounted [1]? Here, the observed differences between task conditions with and without associative memory may provide a quantitative basis for discussion.

Finally, the measures that are compared in this study have been assembled into an “autonomy” python toolbox available at https://github.com/Albantakis/autonomy (accessed on 15 September 2021), which also includes the data set of artificial agents analyzed in this paper. The toolbox allows the application of the various measures to agent objects defined by their transition probability matrices and numbers of sensors, motors and hidden units, as well as other optional features.

## 2. Quantitative Measures Related to Agency, Autonomy, and Intelligence

In the following I will provide an overview across various measures related to intelligence and autonomy compiled across multiple disciplines, including graph-theory, information-theory, and complex system science. These measures are generally applicable to any stochastic system $V=\{V_{1},V_{2},\ldots ,V_{n}\}$ with finite state space $\Omega_{V}$ and current state $v_{t}\in \Omega_{V}$, which is constituted of *n* random variables $V_{i}$ and interacts with an environment *E* with finite state space $\Omega_{E}$ and current state $e_{t}\in \Omega_{E}$. The system is assumed to be Markovian. In that case, the system’s dynamics can be described in terms of its transition probability function

(1) $$p\left(v_{t+1}|v_{t},e_{t}\right)=P\left(V_{t+1}=v_{t+1}|V_{t}=v_{t},E_{t}=e_{t}\right),\;v_{t},v_{t+1}\in \Omega_{V},e_{t}\in \Omega_{E}.$$

The *n* random variables that constitute the system can be divided into sensor (*S*), hidden (*O*), and motor units (*M*). Throughout this study, the state of the sensor units depends only on the environment, while the state of the hidden and motor units depends only on the state of the sensor and hidden units. The motor units thus act on the environment but do not feed back into the system. This strict distinction between hidden and motor units is made for conceptual clarity; none of the measures outlined below depend on it. While the sensor and motor units of an MB thus constitute a Markov Blanket in the traditional, causal sense [20], they are not Markov Blankets as required according to Friston’s free energy principle (FEP) formalism [21], because MBs are not self-organizing (see also [22]).

In line with prior work [1], all measures are formulated under the assumption of discrete states and discrete time for simplicity. While some of the measures could also be extended to more general dynamical systems, for others neural networks with continuous states would have to be discretized appropriately in order to analyze them (see also [23]).

Throughout, upper case letters denote variables, while specific states of a variable or set of variables are denoted by lower case letters.

### 2.1. Structural and Graph-Theoretical Measures

**Figure 1:**
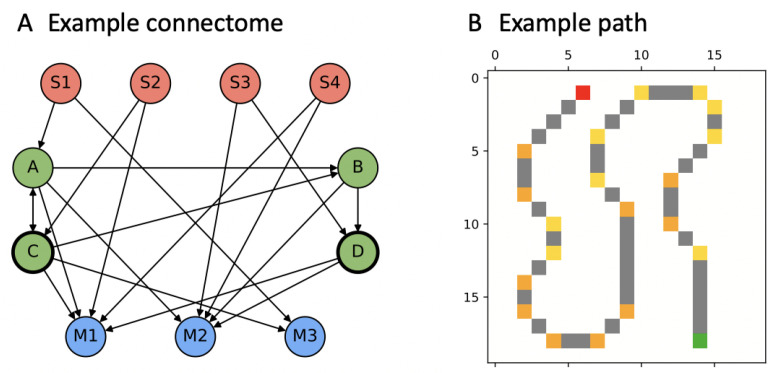
Simulated evolution experiment. (**A**) Example connectome of a Markov Brain (MB) evolved in condition A2 (fitness = 0.92, completion = 1.0, generation = 150,000). The MB has four connected sensors (red), four hidden units (green), and three motor units (blue). Evolutionary optimization determines both the input-output function of each individual node (here binary and deterministic) and the MB connectivity. (**B**) One of the four paths used in the “PathFollow” environment. Green: start location; yellow: left turn symbols; orange: right turn symbols; and red: goal.

While the connectivity structure of artificial neural networks is often externally constrained, this is not the case for Markov Brains (MBs), the type of ANN used in this study (see Figure 1). In this way, MBs more closely resemble biological neural circuits [16]. Assessing the structural features of such systems may thus provide some insight into the demands of a given task environment.

The number of functionally relevant units provides a first simple measure to assess how efficiently a given neural network implements its function and can further be split into the number of connected sensors, motors, and hidden units. To be functional, sensors have to output to other nodes, motors have to receive inputs, and hidden nodes have to receive inputs and output to other nodes in the network.

Similarly, the number of connections between various node classes can be evaluated. The degree-centrality measures the fraction of nodes to which a node in the network is connected and is available in the Python network analysis toolbox “NetworkX” [24] along with numerous other measures to evaluate the structural properties of network graphs. To date, the “autonomy” toolbox incorporates the average degree-centrality, the average betweenness-centrality [25] of all functional hidden nodes, and the flow hierarchy [26] as representative measures on directed graphs. The betweenness-centrality evaluates the sum of the fraction of all-pairs shortest paths that pass through a node. The flow hierarchy is defined as the fraction of edges not participating in cycles in a directed graph.

Finally, the largest strongly connected component (LSCC) may serve as a simple structural measure of integration [27], in line with the notion that an autonomous system must form an integrated whole. Note that, by definition, the LSCC of a feed-forward ANN (fANN) is zero, while it includes the entire set of hidden units for an all-to-all connected recurrent ANN. In the MBs, however, the LSCC may vary across agents and task domains. For completeness, the largest weakly connected component (LWCC) is also included, which may indicate modularity if it is smaller than the total number of functional units.

### 2.2. Information Theoretical Measures

Several recent studies have proposed a connection between information-theoretical properties and the emergence of autonomous (living) systems [1,4,23,28]. Most of these measures can be defined based on the entropy of a probability distribution over a random variable *X*,

(2) $$H\left(X\right)=-\sum_{x\in \Omega_{X}}p\left(x\right)\log_{2}p\left(x\right).$$

The probability distributions evaluated by the information theoretical quantities defined below can be obtained from the observed activity of the agents while performing their tasks. The resulting values thus depend on the accuracy of the sampled probability distributions, as well as the task environments an agent is evaluated in and are thus not intrinsic properties of the agent itself.

A simple measure that is commonly used to quantify the complexity of an agent’s behavior within a given environment is the mutual information (*I*) between its sensors (S) and motors (M) [29,30,31]

(3) $$I_{SMMI}\left(S_{t};M_{t+d}\right)=H\left(M_{t+d}\right)-H\left(M_{t+d}|S_{t}\right)=H\left(M_{t+d}\right)+H\left(S_{t}\right)-H\left(M_{t+d},S_{t}\right),$$

where $H(M_{t+d},S_{t})$ denotes the entropy of the joint probability distribution $p(M_{t+d},S_{t})$. Two agents with identical input-output behavior will necessarily have identical $I_{SMMI}$. A related quantity termed “empowerment” [32] aims to measure how well an agent can perceive its own influence on the environment, defined as the channel capacity between an agent’s actions and subsequent sensor inputs.

The $I_{SMMI}$ is a special case of predictive information $I_{pred}\left(V_{t-1};V_{t}\right)$ [33], and captures how much information about the motor output at time t+d is present in the agent’s sensor state at time *t* (see [34] for an application to an fANN). $I_{SMMI}$ is supposed to be high if the agent efficiently extracts all relevant information from the sensors in order to guide its actions. Note, however, that $I_{SMMI}$ may decrease with increasing fitness in tasks that require memory and also for agents with recurrent architectures that take their own internal state into account to determine the motor output [30,35]. $I_{SMMI}$ may thus decrease with increasing autonomy from the environment. An alternative measure is the predictive information that the system as a whole (*V*) has about its future states

(4) $$I_{pred}\left(V_{t-1};V_{t}\right)=H\left(V_{t}\right)-H\left(V_{t}|V_{t-1}\right)=H\left(V_{t}\right)+H\left(V_{t-1}\right)-H\left(V_{t},V_{t-1}\right).$$

$I_{pred}$, also known as time-delayed mutual information (TDMI) [36,37], can be viewed as the extent to which an agent determines itself [1,38] and has been labeled as the autonomy measure $A^{*}$ [1,4]. However, for agents interacting with their environment, it may be more appropriate to evaluate $I_{pred}$ conditioned on the past *m* states of the environment $\left(E_{t-1},\cdots ,E_{t-m}\right)$, which discounts observed correlations between subsequent system states that are actually due to the environment:

(5) $$A_{m}=H\left(V_{t}|E_{t-1},\cdots ,E_{t-m}\right)-H\left(V_{t}|V_{t-1},E_{t-1},\cdots ,E_{t-m}\right),$$

with m>0 (note that I have shifted the index so that it starts at m=1 as opposed to m=0 in the original formulation. $A_{0}$ in [1] thus corresponds to $A_{1}$ here).

Bertschinger et al. [1] proposed $A_{m}$ as a tentative, quantitative measure of autonomy, but also discuss open issues regarding the mutual influence between the environment and the agent, as well as the problem of identifying the borders of the agent in the first place (see also: [8,18,23,39]).

Here I implemented a version of $A_{m}$ that uses the agent’s sensors *S* in place of the actual environment, as the state of the sensor nodes is set directly by the environment. In that case, the system *V* reduces to {O,M}, the set of hidden and motor nodes:

(6) $$A_{m}^{S}=H\left(O_{t},M_{t}|S_{t-1},\cdots ,S_{t-m}\right)-H\left(O_{t},M_{t}|O_{t-1},M_{t-1},S_{t-1},\cdots ,S_{t-m}\right)$$

In case of deterministic agents, the second part of Equation (6) reduces to zero such that $A_{m}^{S}=H\left(O_{t},M_{t}|S_{t-1},\cdots ,S_{t-m}\right)$.

In [18], Bertschinger and Olbrich also proposed a measure to evaluate a system’s informational closure from the environment. The information flow $J_{t}$ from the environment into the system is defined as the conditional mutual information (*I*) (or transfer entropy [40]) between the current environment state $E_{t}$ and the future system state $V_{t+1}$ given the current system state $V_{t}$:

(7) $$\begin{array}{cc} J_{t}\left(E\to V\right)=I\left(V_{t+1},E_{t}|V_{t}\right) & =H\left(E_{t}|V_{t}\right)-H\left(E_{t}|V_{t},V_{t+1}\right)\\ \; & =H\left(V_{t+1}|V_{t}\right)-H\left(V_{t+1}|V_{t},E_{t}\right). \end{array}$$

$J_{t}\left(E\to V\right)=0$ then indicates informational closure from the environment, which is trivial if the system is independent of the environment and $I(V_{t+1},E_{t})=0$. Consequently, Bertschinger and Olbrich define the non-trivial information closure (NTIC) of a system as

(8) $$NTIC_{m}=I\left(V_{t},E_{t-1},\cdots ,E_{t-m}\right)-I\left(V_{t},E_{t-1},\cdots ,E_{t-m}|V_{t-1}\right)=I_{pred}-A_{m}.$$

NTIC is meant to capture the extent to which the system models its environment [1] and has recently been proposed as a quantity that could be connected to a system’s capacity for consciousness [41]. Note, however, that a large value of NTIC does not ensure a low information flow from the environment into the system and should thus not be considered as a replacement for informational closure, but as a complementary measure [18]. As discussed in [1], NTIC can be negative if the environment and the system jointly determine the next system state. In the “autonomy” toolbox, $J_{t}\left(E\to V\right)$ and $NTIC_{m}$ are again implemented using the agent’s sensor states in place of the actual environment, as for $A_{m}^{S}$ above.

In addition to statistical dependencies and information flows between the system and the environment, the question of when a system “is more than the sum of its parts” lies at the heart of complex system science [42]. This has led to a number of measures of information integration that compare the mutual or predictive information of a system to a partition of the system into two or multiple parts. For recent comparisons of empirical (observational) measures of information integration see [37,42,43,44]. Many of these measures have been conceived as precursory or empirical versions of quantities proposed within the integrated information theory (IIT) of consciousness [45,46,47,48]. Theory-based measures of integrated information (φ and Φ) are, however, intended to be causal, rather than informational measures, meaning they rely on perturbational rather than observed data [46,47,49] and will thus be discussed in the next section.

One simple information measure that captures to what extent the system as a whole (*V*) is more determined than the sum of its parts ($V_{i}$) is the multi-information [50], or total correlation [51], an extension of the mutual information to multiple variables,

(9) $$MI\left(V\right)=\sum_{V_{i}\in V}H\left(V_{i}\right)-H\left(V\right).$$

The multi-information is zero, if and only if all variables $V_{i}$ are mutually independent [42]. An information-theoretic measure developed to capture the capacity of a system for both high local segregation and high global integration is the TSE complexity [52] (named subsequently after the authors of the original publication)

(10) $$C_{TSE}\left(V\right)=\sum_{k=1}^{n}\left(H\left(k,n\right)-\frac{k}{n}H\left(V\right)\right),$$

where H(k,n) is the mean entropy of subsystems of size *k* in the system with *n* elements [52,53]. For an extensive review of proposed multivariate information-theoretical measures of synergy and redundancy see [54].

As a final information-theoretic approach related to agency and autonomy, I want to mention the partial information decomposition (PID) framework [55,56,57,58,59]. The PID framework may be useful in disentangling the contributions of the environment (E) and the system’s own past state in the $A_{m}$ measure listed above, as it allows to determine which part of the information is shared (redundant) between the system and the environment, which information is unique to either, and which part is synergistic [4,39]. The PID framework has been applied to characterize information-theoretical properties in evolved agents [31], and, recently, also to Boltzmann machines [60] and convolutional neural networks CNNs [61]. Mediano et al. [62] recently presented an extension of the PID to multiple target variables to characterize qualitatively different modes of information dynamics.

### 2.3. Causal Measures

The main difference between the causal measures related to autonomy listed below and the information-theoretical measures above is that the causal measures rely on interventional probability distributions instead of observed distributions [1,20,47,63]. Dynamically, a system may not pass through all of its possible states. However, using system perturbations, it is possible to assess how the system reacts when it is set into any of its possible states. Causal measures may thus capture the mechanistic structure of the system in a way that informational measure in general cannot. For example, it is possible to resolve ambiguities in the informational measures that arise due to bidirectional interactions between the agent and the environment through causal interventions [1]. Throughout, the “hat” symbol (^) over variables or operators indicates interventions.

Effective information $EI({\hat{V}}_{t-1},V_{t})$ corresponds to the causal version of $I_{pred}\left(V_{t-1};V_{t}\right)$, assuming a uniform distribution over all system states at t−1 [45,64]

(11) $$EI\left({\hat{V}}_{t-1},V_{t}\right)=|\Omega_{V}{|}^{-1}\sum_{v\in \Omega_{V}}D_{KL}(\hat{p}\left(V_{t}|v_{t-1}\right)\left|\right|\hat{p}\left(V_{t}\right)),$$

where $\hat{p}$ indicates interventional probabilities and $D_{KL}$ denotes the Kullback-Leibler divergence or relative entropy [65]. $EI({\hat{V}}_{t-1},V_{t})$ captures the mechanistic constraints that the system as a whole exerts onto itself, independent of the environment or its observed distribution. $EI({\hat{V}}_{t-1},V_{t})$ is related to ${\hat{A}}^{*}$, a causal measure of autonomy proposed in [1], which also evaluates $I_{pred}\left(\hat{V}\left(t-1\right);V_{t}\right)$. The difference is that for ${\hat{A}}^{*}$ the states of $V_{t-1}$ are perturbed according to their marginal distributions, not maximum entropy as in $EI({\hat{V}}_{t-1},V_{t})$. Along the same lines, Bertschinger et al. [1] also defined a causal version of their $A_{m}$ measure (Equation (5)).

Within the “autonomy” toolbox, I have implemented an intrinsic version of ${\hat{A}}_{m}$ based on the maximum entropy interventional distribution, to remove all dependencies on the dynamics of the environment. For Markovian systems,

(12) $${\hat{A}}_{m}^{S}=H\left(O_{t},M_{t}|{\hat{S}}_{t-1},\cdots ,{\hat{S}}_{t-m}\right)-H\left(O_{t},M_{t}|{\hat{V}}_{t-1}\right).$$

In deterministic systems, the second term reduces to 0 as in Equation (6). Because the sensor states at t−m are set to maximum entropy, ${\hat{A}}_{1}^{S}=EI\left(V_{t},{\hat{V}}_{t-1}\right)$.

While $EI({\hat{V}}_{t-1},V_{t})$ evaluates the system as a whole, the causal framework of integrated information theory (IIT) [47,48,66] aims to characterize the compositional causal structure of a system [67]. The main quantity, Φ (“big phi”), measures to what extent the system “exists for itself” in causal terms, above a background of influences from the environment [3,8]. Within a larger system, the system subset with the largest Φ value is called the “major complex”. For feed-forward systems Φ=0 by construction. This is because, according to IIT, a system only forms an integrated whole if each part of the system has irreducible causes and effects on the rest of the system, alone or in combination. As the ANNs used in this study only have recurrent connections among their hidden units, the maximal possible size of the major complex corresponds to the number of hidden units of the agent.

Regardless of a system’s architecture, the number of internal mechanisms (subset of system elements with positive integrated information φ (“small phi”)) and the sum of their φ values (∑φ) provide a measure of the system’s compositional causal complexity [30,68]. φ captures how much a set of elements *Y* within the system in its current state $y_{t}$ constrains the system’s previous and next states. In simplified terms,

(13) $$\varphi \left(y_{t}\right)=\min_{t\pm 1}\left(\varphi (y_{t},\psi^{*},Z_{t\pm 1}^{*})\right)=\min_{t\pm 1}\left(D\left(\frac{\hat{p}\left(Z_{t\pm 1}^{*}|y_{t}\right)}{{\hat{p}}^{\psi^{*}}\left(Z_{t\pm 1}^{*}|y_{t}\right)}\right)\right),$$

where $\psi^{*}$ partitions $\left(Z_{t\pm 1}^{*}|y_{t}\right)$ into a product distribution $\left(Z_{1,t\pm 1}^{*}|y_{1,t}\right)\times \left(Z_{2,t\pm 1}^{*}|y_{2,t}\right)$, *D* is the difference measure between the two interventional probability distributions, and the * subscript indicates an optimization over system subsets $Z_{t\pm 1}\subseteq V$ and a minimization over possible partitions ψ. A complete description of the φ measure according to “IIT 3.0” can be found in [47,69]. For an updated account that features a new, intrinsic difference measure, see [49].

IIT’s causal analysis evaluates the intrinsic constraints a system exerts onto itself. Recently, we have developed an accompanying account of actual causation (AC) (“what caused what”) [70] to identify the actual causes of an agent’s actions and quantify their causal strength ($\alpha_{c}$). Again in simplified terms,

(14) $$\alpha_{c}\left(x_{t-1},y_{t}\right)=\log_{2}\left(\frac{\hat{p}\left(x_{t-1}|y_{t}\right)}{{\hat{p}}^{\psi^{*}}\left(x_{t-1}|y_{t}\right)}\right),$$

where $x_{t-1}\subseteq v_{t-1}$ and $y_{t}\subseteq v_{t}$ are system subsets whose state is determined by the transition $v_{t-1}≺v_{t}$ of the system from t−1 to *t*, and $\psi^{*}$ denotes a minimal partition of the link between $x_{t-1}$ and $y_{t}$. The causal strength $\alpha_{c}\left(x_{t-1},y_{t}\right)$ can be viewed as the irreducible causal information that an occurrence $y_{t}$ specifies about a possible cause $x_{t-1}$ (see also [71]). The actual cause $x_{t-1}^{*}$ of $y_{t}$ is the one that maximizes $\alpha_{c}\left(x_{t-1},y_{t}\right)$, such that $\alpha_{c}\left(y_{t}\right)=\alpha_{c}\left(x_{t-1}^{*},y_{t}\right)=\max_{x_{t}}\left(\alpha_{c}\left(x_{t-1},y_{t}\right)\right)$. For a rigorous definition of $\alpha_{c}$ based on product probability distributions, see the original publication [70].

As shown in [72], the AC framework also makes it possible to trace the causes of an agent’s action back in time (“causes of causes”) and to evaluate the relative causal contributions of an agent’s internal mechanisms and states to its actions. Specifically, the average contribution of the hidden units *O* at time t−1 to the actual causes of the motor units *M* being in state $m_{t}$ can be quantified as

(15) $${\bar{\alpha }}_{c}\left(O≺M\right)=\sum_{y_{t}\subseteq m_{t}}\frac{S_{O}\left(\alpha_{c}\left(y_{t}\right)\right)}{\alpha_{c}\left(y_{t}\right)},$$

where $S_{O}\left(\alpha_{c}\left(y_{t}\right)\right)$ is the Shapely value [73] of $o_{t-1}\cap x_{t-1}^{*}$, the subset of hidden units *O* in the actual cause of $y_{t}\subseteq m_{t}$. The Shapely values are evaluated with $\alpha_{c}$ (Equation (14)) as the value function. Note that the measure is compositional: the actual causes of all subsets of $y_{t}\subseteq m_{t}$ are taken into account.

Finally, it is important to emphasize that all IIT derived measures are state-dependent. It is thus possible to assess their variability within an agent and also to evaluate the agent at various points in time [72,74] (see [31,39] for state-dependent versions of some of the above-listed information-theoretical measures). To obtain values for individual agents, the state-dependent quantities are averaged across all states (for 〈∑φ〉 and $\langle \Phi^{\max}\rangle$) or state transitions (for $\langle {\bar{\alpha }}_{c}\left(O≺M\right)\rangle$) weighted by their probability of occurrence.

All IIT quantities can be computed with PyPhi, a python toolbox developed by the Tononi lab [69]. The “autonomy” toolbox also contains all causal measures described and assessed in this study.

### 2.4. Dynamical Measures

Dynamical systems theory is concerned with characterizing the behavior of complex systems over time [31,75]. Two main types of analysis can be distinguished: first, one can assess the dynamical complexity of a system’s trajectories. Second, a system’s long-term behavior may be analyzed to determine how quickly the system settles into a dynamical fixed point or limit set.

Characterizing the long-term behavior of open systems is complicated by the fact that their dynamics are constantly perturbed by the environment. Nevertheless, it is possible to evaluate the average and maximum transient length before an agent converges to a steady state if its sensor inputs are kept constant through perturbation [68].

Possible quantifiers of dynamical complexity include the morphological diversity [76] and information or compression based approximations of the (incomputable) Kolmogorov complexity (KG) of the systems’ dynamical transients [77,78,79]. Morphological diversity measures the number of distinct square patterns in a system’s evolution. As the MBs investigated here have no explicit topological structure, I will focus instead on a simple compression based measure of KG, the normalized Lempel–Ziv complexity (nLZ) [77]. LZ-complexity measures have been applied to investigate the functional complexity of cellular automata and small neural networks [80,81], and also to neurophysiological recordings to assess the level of consciousness in human subjects [82].

To quantify nLZ, an agent’s activity data is first converted into a one-dimensional string of binary symbols. Here, activity data is generally formatted as a two-dimensional array at the start, with different time steps along one dimension and system units along the other. The two-dimensional array may thus be reshaped in time ([$V_{1,t}$, ⋯, $V_{1,t+m}$; ⋯; $V_{n,t}$, ⋯, $V_{n,t+m}$]) or space [$V_{1,t}$, ⋯, $V_{n,t}$; ⋯; $V_{1,t+m}$, ⋯, $V_{n,t+m}$]. The LZ-complexity of the resulting one-dimensional bit-string corresponds to the number of unique “words” of any length within the string. To account for biases in the number of binary symbols and their entropy, this value is then normalized by the average LZ-complexity of a number of random permutations of the original data string (in the limit of infinite strings, this normalization factor converges to the string’s bit entropy [82]).

Similar to the choice between observed or perturbational probability distributions that distinguishes informational from causal measures above, one can evaluate the dynamical complexity of an agent’s recorded activity while performing the task, or its dynamical transients upon perturbation.

This leaves four nLZ measures to be evaluated: nLZ_time and nLZ_space are based on an agent’s recorded activity while performing the task, while nLZ_tr_time and nLZ_tr_space are based on an agent’s dynamical transients upon perturbation into all possible states while holding the sensors fixed. The “time” label refers to a reshaping of the activation data in time, as described above; likewise for “space”.

The “autonomy” toolbox, moreover, allows evaluating the number of unique transitions in an agent’s recorded activity, a simple measure that may be indicative of an agent’s dynamical complexity [72].

## 3. Evolution Simulation

### 3.1. Markov Brains (MBs)

MBs are a class of evolvable artificial neural networks. Their main difference from conventional ANNs is that instead of a layered architecture, with each node performing the same function, MBs are networks built from individual computational components (“neurons”, here limited to binary gates with generalized logic functions) [16]. These computational components interact with each other, receive inputs from sensors, and control motor outputs (Figure 1). The connectivity and input-output function of each neuron is, moreover, subject to evolutionary optimization. Here, MBs are genetically encoded and evolve through mutation and selection at the level of the genotype.

Software to evolve artificial agents controlled by MBs (and other types of ANNs) in various task environments is freely available as part of the “MABE” (Modular Agent Based Evolver) framework [83]. This study employed https://github.com/Hintzelab/MABE/commits/development,commit 834b5b0ea8c3b69ebfeb9c7ecebdb20f726c71f1. (accessed on 2 August 2021) Agents were limited to four hidden units and deterministic gates with zero initial gates at the beginning of their evolution. Standard settings were used for the evolution optimization and genome encoding. Dynamically, MBs are fully described by their transition probability matrix (Equation (1)), which can be obtained from MABE using the “TPM_world” environment.

Even small, binary, deterministic implementations of MBs are capable of achieving high performance across a variety of tasks, such as spatial navigation (e.g., passing through mazes) [29], active perceptual categorization tasks [30,35], or interactive tasks with multiple agents [27,84,85]. Since the connectivity structure of a MB is evolved, the degree to which it is feed-forward, modular, or recurrent depends on the specific task environment and chance (or rather, the random seed). As previously demonstrated, animats that evolved to more difficult task environments, which required greater context-sensitivity and internal memory, tended to develop MBs with more recurrent network architectures, stronger intrinsic causal constraints and higher information integration Φ [30]. However, a greater number of sensors may offset internal complexity by alleviating the need for memory in certain task conditions [30,85].

### 3.2. “PathFollow” Environment

At every generation in the evolution optimization, agents were evaluated within their task environment. In this study, MBs were trained to solve a spatial navigation task, MABE’s “PathFollow” world, which was first described in [15] as an associative memory task (see also https://github.com/Hintzelab/MABE/tree/feature-path_follow_world/code/World/PathFollowWorld, accessed on 15 September 2021). In this task, the agent is rewarded for each location visited along a predefined path, with $45^{\circ }$ turns indicated by left and right turn symbols (Figure 1B). If an agent reaches the goal before the time out (number of path locations plus 50 extra steps), the remaining time is added to the number of visited locations in the fitness function. Finally, there is an “empty space cost” of −0.25 for every step the agent takes off the path, which explains the initial negative fitness values.

The data set analyzed in this study consists of 50 independent evolution simulations under 3 task conditions that differ in the number of symbols that could indicate a left or right turn (see Table 1). In the simplest condition (“NA”), the left and right turn symbols are fixed across trials and generations (“0” for left and “1” for right). This condition does not require associative memory within a trial. The second condition (“A2”) had the same two turn symbols, but their meaning (left or right) was randomly assigned in each trial (for each evaluated path). This required the agents to identify and store the correct association. The third condition (“A4”) included four random turn symbols, (“00, 01, 10, 11”). In addition, agents received bit-wise inputs about whether they are on path (S1), off path (S2), or on a turn location (S3). The remaining sensors encode the turn symbols (one for NA and A2, and two for A4). All agents were equipped with three motor outputs, with 000 = no movement, 100 = left, 010 = right, 110 = forward, XX1 = reverse (X = 0 or 1).

**Figure 2:**
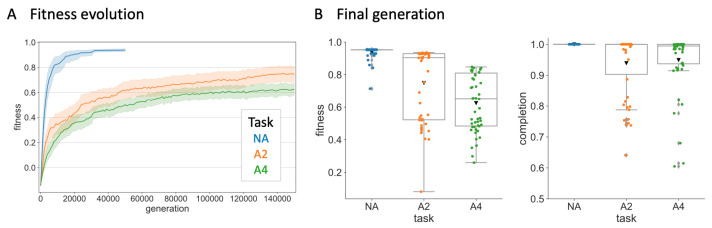
Fitness evolution and distribution across task conditions. (**A**) Fitness evolution across number of generations. Shaded area indicates 95% confidence interval. (**B**) Distribution of fitness values (left) and percentage of path completion (right) in the final generation. Black triangles indicate mean. Perfect completion was achieved by 50/50 MBs in NA, 31/50 in A2, and 16/50 in A4.

Agents were tested on each of the four paths included in the MABE PathFollow world and their flipped versions. In the case of random turn symbols (A2 and A4) the task evaluation was repeated 10 times per generation to reduce variation in the fitness values across generations. To ensure independent samples, one agent was chosen from the final generation in each evolution simulation. For each of these agents it is possible to trace back their line of descent (LOD) (there is no recombination in this evolution simulation). Figure 2 shows the average fitness evolution across the 50 LODs for each of the three task conditions. Note that A2 and A4 agents required more time steps to solve the task, as they first have to identify the correct turn symbols, or use compensatory strategies. This explains their lower fitness values even for full path completion.

### 3.3. Data Analysis

Pearson correlation coefficients were evaluated between selected measures using the “scipy.stats” Python package. All reported correlation coefficients were highly significant with $p\ll 1.0\times 10^{-10}$. Appendix A includes a correlation matrix of all evaluated measures.

## 4. Results

As the goal is to compare agents with similarly high task performance but different network structures, the subsequent analysis is focused on the subset of evolved MBs (final generation) that completed all of the training maps (50/50 in NA, 31/50 in A2, and 16/50 in A4). To ensure that the resulting MBs were not overfitted to the particular maps they were evolved to, all MBs with full completion were successfully tested on a separate set of two test maps. Note however, that completing the maps does not necessarily require associative memory in condition A2 or A4, as other, compensatory strategies may be successful, albeit at a cost of extra time steps. A detailed analysis of the agents’ various evolved behavioral strategies will be presented in a companion paper.

### 4.1. Evolved Network Structures

**Figure 3:**
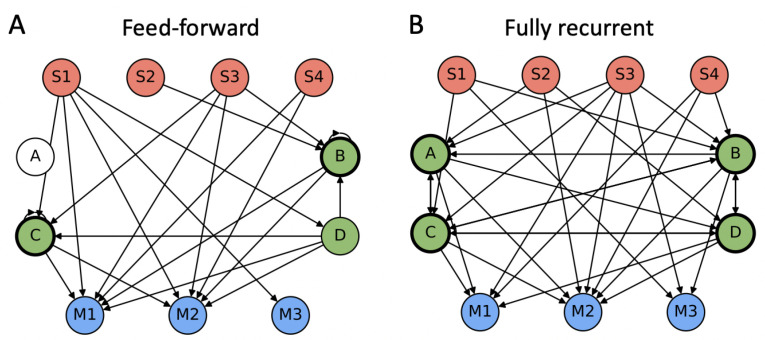
Example connectomes of two A2 MBs with perfect completion, but feed-forward or fully recurrent connectivity, respectively. (**A**) MB with only feed-forward connections between units, although nodes B and C have self-loops. Thus, the length of the LSCC is one for this MB. (**B**) MB with recurrent connections between all hidden units and largest possible LSCC length of four hidden units.

Out of the 97 agents that completed all paths, all but two MBs in condition NA used all their motor units (the two remaining agents lacked the capacity to move backwards). By contrast, only 10/50 NA agents and 21/31 A2 agents used all of four available sensors, which can be explained by the redundancy in input information in the first three sensors. In the A4 condition, all MBs lacked at least one available sensor and notably either S4 or S5, which encode the turn symbols. What is more, 2/16 A4 agents evolved to complete the paths without relying on turn symbols at all, as they lacked both S4 and S5. The average number of connected hidden units was lower in NA (1.9±0.6) than in A2 (3.4±0.5) and A4 (3.4±0.5), consistent with a need for more hidden units in the more demanding task conditions. See Figure 3 for two example connectomes.

**Figure 4:**
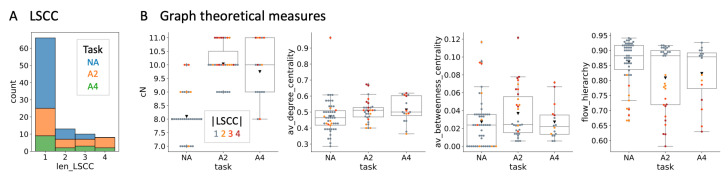
Structural analysis. (**A**) Stacked histogram of the LSCC length for the three task conditions. While most MBs in the NA condition are feed-forward (len_LSCC = 1), both feed-forward and recurrent architectures evolved in all three task conditions. (**B**) Distributions of the number of connected nodes (cN), average degree centrality, average betweenness centrality, and flow hierarchy are shown across task conditions and color-coded according to the length of their LSCC. MBs evolved in A2 and A4 were larger than those in NA by approximately two nodes. The other graph-theoretical measures show little difference between task conditions. As the flow hierarchy depends on cyclical connectivity, lower values correspond to MBs with larger LSCCs. Please note that throughout, axis labels correspond to variable names assigned to the various measures in the accompanying autonomy toolbox.

The structural measure most directly related to autonomy is the length of the LSCC of a MB (Figure 4A). Many have argued that an autonomous system must form a unified whole that can be regarded as separate from the environment (yet interacts with it) [3,6,7,8,9,10]. Interpreted in structural terms, this would imply that the system must be strongly connected in its network architecture, which means that every node (causally) connects to every other node on a directed path. Feed-forward ANNs do not fulfill this condition, since it requires recurrent connectivity.

The MBs evolved in this study have at most four hidden units that could be strongly connected (sensors and motors are connected in a feed-forward manner). In the following, I will distinguish between MBs that have a subset of at least two strongly connected hidden units and MBs with only feed-forward connections between units (Figure 3).

Specifically, the length of the LSCC of a MB will be indicated by color as in Figure 4B, to highlight whether or not a particular measure depends on network architecture and in which way. None of the 97 agents are connected in a purely feed-forward manner, as they all have at least one hidden unit with a self-loop. Nevertheless, feed-forward and recurrent network architectures (between nodes) can be found in all task conditions in the set of MBs with full completion.

Figure 4B provides a summary of the structural properties of the evolved MBs for the three different task conditions. While A2 and A4 MBs had approximately two more nodes than NA MBs, more complex graph-theoretical measures did not differ much between conditions.

### 4.2. Information Theoretical Analysis

**Figure 5:**
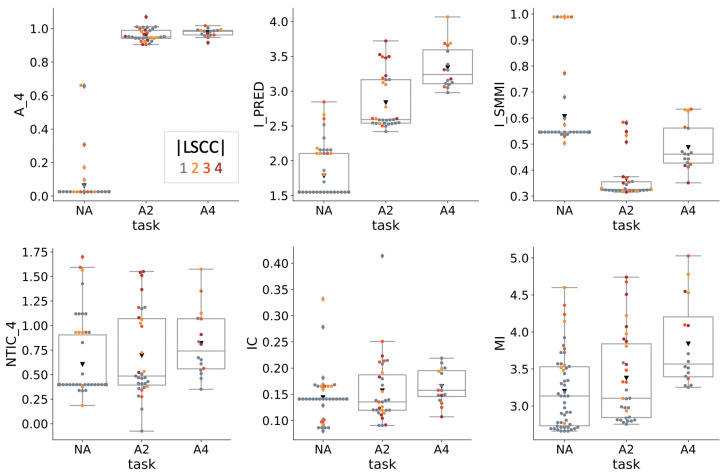
Information-theoretical analysis. The complimentary measures of autonomy proposed in [1], $A_{4}$ and $I_{pred}$, as well as $I_{SMMI}$ identify significant differences across task conditions (top row). By contrast, the information closure measures, $NTIC_{4}$ and $J_{t}$ (here “IC”) (bottom row) do not differ much between conditions. The multi-information (MI) is higher for A4, than the other two conditions, with higher values for MBs with len_LSCC >1.

Figure 5 summarizes the information-theoretical properties of the evolved MBs. The two complimentary measures of autonomy proposed in [1], $A_{m}$ (Equation (5)) and $A^{*}\;=\;I_{pred}$ (Equation (4)) are shown in the first row, in addition to $I_{SMMI}$ (Equation (3)).

$A_{m}$ was evaluated over four time steps of sensor inputs as $A_{4}^{S}$ according to Equation (6). Notably, $A_{4}^{S}$ shows very little variance within task condition. While $A_{4}^{S}$ remains close to zero in NA, it detected approximately 1 bit of autonomous information in A2 and A4. In condition A2, this bit likely corresponds to the associative memory, the internal representation of the turn symbol encoding (whether S4 = 1 means right or left in a given trial). If A4 agents would solve the “PathFollow” task relying on associative memory alone, one might expect a higher value of $A_{4}^{S}$ in A4 than in A2. However, none of the A4 MBs actually uses both task symbol sensors. Overall, $A_{m}$ seems to reflect task demands, rather than agent specific properties, such as the MBs particular implementation or structural properties of individual MBs, at least in this particular simulation experiment.

Mean values of $A^{*}=I_{pred}$ differ significantly between all three task conditions. In all three conditions, the highest values of $I_{pred}$ were achieved by MBs with len_LSCC >1. Moreover, since the MBs used in this study are deterministic, $I_{pred}$ is almost perfectly correlated with system entropy *H* (ρ=0.99) (see Appendix B). The correlation between $A_{4}^{S}$ and $I_{pred}$ is high at ρ=0.85, despite the low within-task variance of $A_{4}^{S}$. The $I_{SMMI}$, here evaluated across one time step (d=1 in Equation (3)), is lowest in condition A2, in line with the observation that internal memory decreases this measure.

The two complimentary measures of informational closure, $NTIC_{m}$ (Equation (8)) and $J_{t}$ (Equation (7), “IC” in Figure 5) are shown in the second row of Figure 5, together with the multi-information MI (Equation (9)). Like $A_{m}$, $NTIC_{m}$ was computed over four time steps of sensor inputs (m=4). Differences between task conditions for these measures are small. MI is larger in A4 than the other two measures. As a “whole-minus-sum” entropy measure, MI is correlated with $C_{TSE}$ (ρ=0.77, see Appendix B). Moreover, MBs with larger LSCCs have higher values of MI and $C_{TSE}$ across task conditions.

### 4.3. Causal Analysis

**Figure 6:**
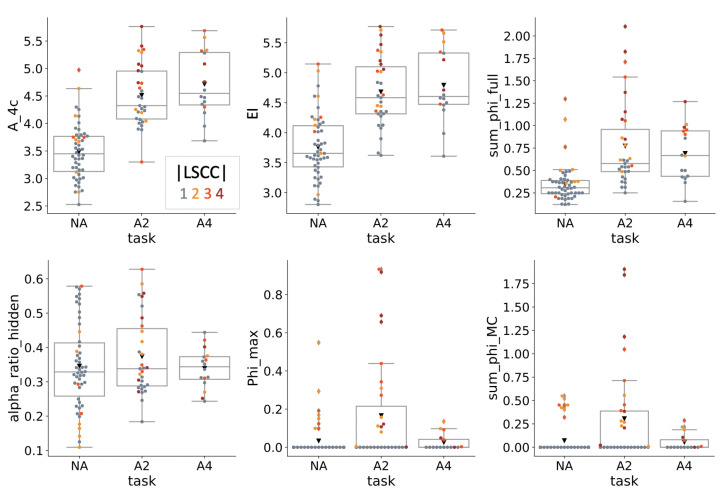
Causal analysis. The top row shows the causal version of the autonomy measures proposed in [1], ${\hat{A}}_{4}$ and $EI(V_{t},V_{t-1})$, as well as 〈∑φ〉 evaluated for the whole MB including sensors and motors. Note however, that here ${\hat{A}}_{4}$ (“A_4c”) and $EI(V_{t},V_{t-1})$ are based on a maximum entropy distribution of input states rather than the marginal observed distribution proposed in [1]. For all three measures, the NA condition had lower values than A2 and A4. The bottom row shows $\langle {\bar{\alpha }}_{c}\left(O≺M\right)\rangle$, the relative contribution of the hidden units (*O*) to the actual causes of the agent’s motor states (“alpha_ratio_hidden” in the figure), together with $\langle \Phi^{\max}\rangle$ and ${\langle \sum \varphi \rangle }_{MC}$ values of the major complex (the maximally integrated subset of hidden units). $\langle {\bar{\alpha }}_{c}\left(O≺M\right)\rangle$ values vary substantially within task condition rather than across conditions, which indicates a large variety of behavioral strategies within each task condition. While condition A2 on average has higher values of $\langle \Phi^{\max}\rangle$ and ${\langle \sum \varphi \rangle }_{MC}$ than NA and A4, these IIT measures are zero by definition for MBs with len_LSCC <2 and, in general, depend strongly on implementation.

The results of the causal agent analysis are summarized in Figure 6. ${\hat{A}}_{m}$, the causal version of $A_{m}$ [1], was evaluated over four time steps of sensor inputs as ${\hat{A}}_{4}^{S}$ (Equation (12)) for comparison with the corresponding information theoretical quantity $A_{4}^{S}$ in Figure 5, which was based on the agents’ observed distributions. By contrast to $A_{4}^{S}$, the causal version ${\hat{A}}_{4}^{S}$ shows substantial variation within task condition, but is still higher for A2 and A4 MBs than for NA MBs. The correlation between ${\hat{A}}_{4}^{S}$ and $A_{4}^{S}$ is ρ=0.70.

The effective information $EI(V_{t},V_{t-1})$ (Equation (11)) is equivalent to ${\hat{A}}_{1}^{S}$ and is also related to ${\hat{A}}^{*}$ as proposed in [1], but imposes a maximum entropy distribution on input states instead of the marginal observed distribution. The small differences between $EI(V_{t},V_{t-1})$ and ${\hat{A}}_{4}^{S}$ are thus due to the additional number of past sensor states taken into account for ${\hat{A}}_{4}^{S}$ in Equation (12). The correlation between $EI(V_{t},V_{t-1})$ and ${\hat{A}}_{4}^{S}$ is ρ=0.93. $EI(V_{t},V_{t-1})$ is also correlated to $I_{pred}$ (ρ=0.78), which is based on the observed distribution of the recorded activity.

The higher values of ${\hat{A}}_{4}^{S}$ compared to $A_{4}^{S}$, and also of $EI(V_{t},V_{t-1})$ compared to $I_{pred}$ can be explained by the higher entropy of the perturbational input distributions compared to the observed distribution (Appendix B), but also reflect a stronger causal influence between subsequent internal states than can be observed through correlation.

〈∑φ〉 evaluates the compositional causal structure of the MBs, including sensor and motor units. It is higher for A2 and A4 than NA, and correlates strongly with len_LSCC (ρ=0.80).

$\langle {\bar{\alpha }}_{c}\left(O≺M\right)\rangle$ measures the relative contribution of the agent’s hidden units (*O*) to the direct actual causes of its motor outputs. As shown in Figure 6 (“alpha_ratio_hidden”), $\langle {\bar{\alpha }}_{c}\left(O≺M\right)\rangle$ varies substantially within task condition. This indicates that the various MBs evolved to solve the “PathFollow” task do so using a variety of different implementations and behavioral strategies in all three test conditions. Moreover, $\langle {\bar{\alpha }}_{c}\left(O≺M\right)\rangle$ seems to highlight an aspect of autonomous behavior that is not captured by any of the other proposed measures of autonomy, since they do not correlate with $\langle {\bar{\alpha }}_{c}\left(O≺M\right)\rangle$ (see Appendix A).

The two remaining IIT-based measures, $\langle \Phi^{\max}\rangle$ and ${\langle \sum \varphi \rangle }_{MC}$ are based on the major complex within a given MB, which corresponds to the maximally integrated subset of hidden units (evaluated here according to “IIT 3.0” [47,69]). These measures are zero by definition for feed-forward MBs with len_LSCC <2, since these types of networks do not have any integrated subsets. Of those agents with len_LSCC >1, A2 agents achieved the highest values of $\langle \Phi^{\max}\rangle$ and ${\langle \sum \varphi \rangle }_{MC}$.

### 4.4. Dynamical Analysis

**Figure 7:**
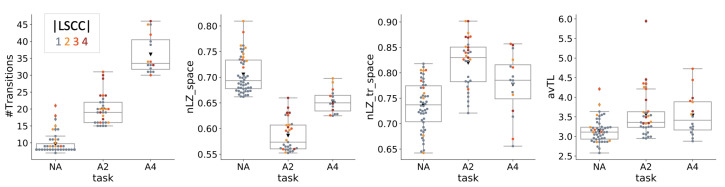
Dynamical analysis. The first panel shows the number of unique transients per task condition while performing the task. The middle two panels show the normalized Lempel-Ziv complexity of the MBs’ recorded activity (nLZ_space) and the MBs’ transients upon perturbation into all possible initial states for fixed sensor inputs (nLZ_tr_space). Notably, the ordering of nLZ for recorded activity patterns (nLZ_space) across conditions is reversed under perturbation (nLZ_tr_space). Average transient length (avTL) is larger for A2 and A4 than NA.

Figure 7 shows the results of the dynamical agent analysis. Based on the recorded activity of each MB performing the “PathFollow” task, I evaluated the number of unique transitions and the normalized Lempel-Ziv complexity (nLZ) (Figure 7, first two panels, shown is the nLZ for a spatial reshaping of the activity data to one dimension). The number of unique transients differs significantly between task conditions and is strongly correlated with entropy *H* (ρ=0.93). Perhaps surprisingly, the activity based nLZ_space is strongly correlated with $I_{SMMI}$ (ρ=0.79). A similar pattern across task conditions was also found for nLZ_time, but with smaller differences between conditions (see Appendix B).

Notably, the normalized Lempel-Ziv complexity of the MBs’ transients upon perturbation into all possible states for fixed sensor inputs (nLZ_tr_space), shows the opposite ordering of task conditions, being highest in A2. Again, the temporally ordered nLZ upon perturbation (nLZ_tr_time) behaved analogously to the spatially ordered nLZ, with smaller differences between A2 and A4 (see Appendix B).

The difference between the activity-based and perturbational nLZ results suggests that the dynamical complexity of the NA condition is almost fully accounted for by the environment, whereas the potential for dynamical complexity of A2 and A4 agents is not exhausted during task performance.

Finally, the average transient length (avTL) is higher for A2 and A4 agents and correlates strongly with the maximum transient length (mTL) (ρ=0.87, see Appendix B).

## 5. Discussion

Autonomy means self-determination. Nevertheless, it has been emphasized repeatedly that our notion of autonomy is in fact multi-dimensional, comprising multiple aspects, and may be evaluated across various domains [1,2,5,12,13,14]. For example, Moreno et al. [12] distinguish between “interactive” (cognitive) and “constitutive” (biological) autonomy; Boden [2,86] highlights three aspects: how much a system’s response to the environment is mediated by internal mechanisms (self-determination), the extent to which these internal mechanisms are self-generated (self-generation), and whether they are flexibly modifiable from within (self-modification); Vakhrameev et al. [5] propose to distinguish self-generation, self-organization, and self-control. It is thus not surprising that different measures focus on different aspects when it comes to determining whether a system qualifies as an autonomous agent.

**Figure 8:**
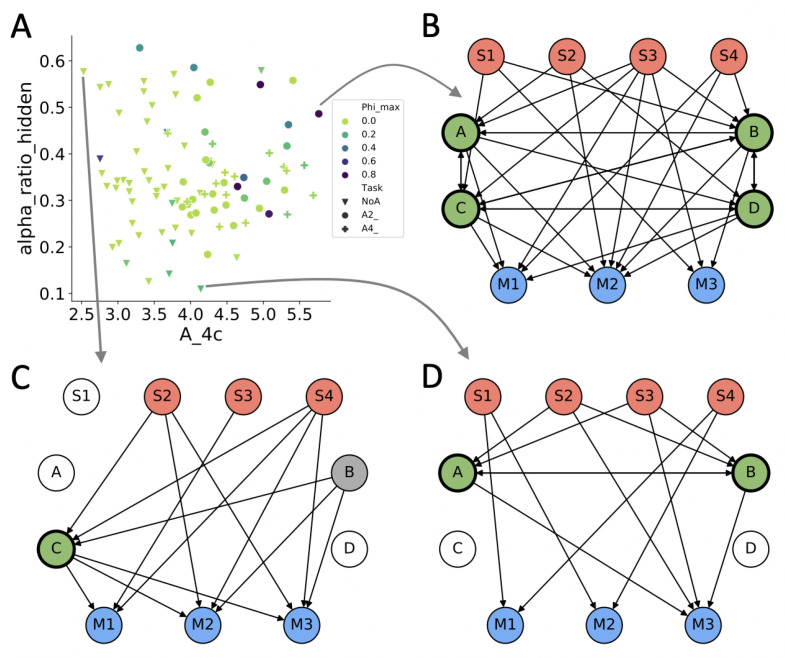
Example networks with different amounts of autonomy. (**A**) The scatter plot of $\langle {\bar{\alpha }}_{c}\left(O≺M\right)\rangle$ (alpha_ratio_hidden) against ${\hat{A}}_{4}^{S}$, color-coded by the amount of $\langle \Phi^{\max}\rangle$ compares three causal measures of autonomy that represent agency, self-determination, and causal closure, respectively. (**B**) Connectome of A2 MB with high values for three orthogonal measures of autonomy, $\langle {\bar{\alpha }}_{c}\left(O≺M\right)\rangle$, ${\hat{A}}_{4}^{S}$, and $\langle \Phi^{\max}\rangle$. (**C**) Connectome of NA MB with low ${\hat{A}}_{4}^{S}$ and $\langle \Phi^{\max}\rangle =0$, but high $\langle {\bar{\alpha }}_{c}\left(O≺M\right)\rangle$. (**D**) Connectome of NA MB with low $\langle {\bar{\alpha }}_{c}\left(O≺M\right)\rangle$, but intermediate ${\hat{A}}_{4}^{S}$ and $\langle \Phi^{\max}\rangle$.

The structural, information-theoretical, causal, and dynamical measures related to autonomy compared above, fall into three conceptual categories: (I) self-determination (how much the system determines its own internal states), (II) closure (whether the system forms an independent entity above a background of external influences), and (III) agency (whether and to what extent the actions of the system are determined by its internal mechanisms, as opposed to external influences) (Figure 8). Self-determination is captured in different ways by $A_{m}$, $I_{pred}$, ${\hat{A}}_{m}$, $EI(V_{t},V_{t-1})$, as well as nLZ evaluated based on perturbational transients. To what extent a system is structurally, informationally, or causally closed is evaluated by len_LSCC, informational closure $J_{t}$, $NTIC_{m}$, and $\langle \Phi^{\max}\rangle$, respectively. In addition, MI and $C_{TSE}$ evaluate whether a system is more than the sum of its parts in information-theoretical terms. While I have listed the integrated information $\langle \Phi^{\max}\rangle$ of a system [47] as a measure of causal closure, it also requires causal self-determination and captures the notion of a system “being more than the sum of its parts”. Specifically, Φ quantifies how much the various parts of a system constrain each other alone and in combination, irreducibly, above a background of external influences. Arguably, any system with $\langle \Phi^{\max}\rangle >0$ thus possesses some amount of self-determination, closure, and self-organization [5]. Finally, $\langle {\bar{\alpha }}_{c}\left(O≺M\right)\rangle$ captures to what extent an agent’s actions are (directly) caused from within.

In the following, I will (1) briefly outline the scope and limitations of this study, (2) highlight related work, including several measures not included in the comparison above, (3) question the tension between memory and self-determination that underlies some of the evaluated measures, and (4) discuss conceptual differences between information-theoretical and causal measures of autonomy.

### 5.1. Scope and Limitations

The artificial agents evaluated in this study correspond to minimal cognitive systems, whose neural architecture and functionality evolved across generations, but remains fixed within each particular generation. Therefore, this work does not address issues related to constitutive self-generation, self-maintenance, metabolism, or autopoiesis [87,88,89], although some of the measures reviewed above may be applied to a dynamical description of the self-maintaining processes of a biological or artificial organism [4,8,18]. For similar reasons, the relation between autonomy and the thermodynamical properties of a system [4,12,23,90,91] lies outside the scope of this study (although the types of causal networks defined by Equation (1) are conceived as approximations of physical systems).

An example of an information-theoretical framework related to autonomy that relies on self-maintenance is Friston’s free energy principle (FEP) formalism, which requires the system to be ergodic [21]. While it may still be possible to translate these ideas to the type of small systems employed here [19,92], the FEP connects the statistical boundaries of a system, its Markov Blanket, with an optimality principle, the minimization of information-theoretical free energy over time. However, the only optimization process the MBs are undergoing is their evolution. Whether and in which way the FEP formalism can be meaningfully applied to characterize the autonomy or behavior of an MB-like automaton with a fixed TPM is an important issue to be addressed in future work. Identifying minimal computational systems in which principles of IIT and FEP can be compared directly could greatly elucidate points of similarity and divergence between the two frameworks (see [92,93,94,95]).

### 5.2. Related Work

Several studies have compared subsets of the measures compiled in this study. Beer and Williams [31] compared the utility of information-theoretic and dynamical measures for understanding the behavior of an evolved artificial agent. Timme et al. [54] reviewed multivariate information measures of synergy and redundancy and applied them to small computational and neural systems. Kanwal et al. [42] compared several information theoretic measures of complexity in Boltzmann Machines. Multiple recent studies [37,43,44,81] compared proposed empirical measures of information integration in small neural networks. Previous studies on adapting animats have evaluated the evolved MBs under a variety of structural, information-theoretic, and causal measures [29,30,35,68]. However, a systematic comparison of multi-disciplinary measures related to autonomy and intelligent behavior of the scope presented in this study has not been conducted to date.

Nevertheless, the list of structural, information-theoretical, causal, and dynamical measures assembled in the “autonomy” toolbox and compared above is not exhaustive and can be expected to grow further. For example, Marstaller et al. [35] introduced an information-theoretical measure of representation, which quantifies the shared entropy between representative features of the environment and the agent’s internal states given its sensor states. However, *R* can be difficult to interpret depending on the way in which the sensor information is processed within the rest of the system.

Another type of causal analysis has been proposed by Shalizi and Crutchfield [96] within their computational mechanics framework. Their goal is to identify the “ϵ-machine” of a statistical process (or the transient of a dynamical system), which corresponds to the minimal causal-state representation of that system consistent with accurate prediction. In deterministic, Markovian systems, such as the MBs investigated here, the ϵ-machine is determined by the number of unique rows in the system’s transition probability matrix, and thus related to $EI(V_{t},V_{t-1})$ (Equation 11) and a measure of the system’s differentiation proposed in [97], which is also related to the viability function proposed by Kolchinsky and Wolpert [23].

Additional candidate measures include the local information framework [98,99], as well as other causal/perturbational measures such as local sensitivity [100], which so far have mainly been applied to a notion of agents based on persistent spatio-temporal patterns [101,102], rather than systems of interacting mechanisms such as artificial neural networks (ANNs). It remains to be determined how these and other measures related to autonomy may be applied to ANNs.

In general, the objective in current AI research is performance optimization. While efficiency plays a role with respect to available computational resources, the internal structure or specific functionality of a high-performing ANN is otherwise of little concern. For this reason, and because qualitatively different network architectures excel in distinct task domains, comparisons between different types of networks performing the same tasks are rare. One exception is recent work by Hintze et al. [103], who compared the evolved representation (R) and its “smeardness” across hidden units in MBs and rANNs performing the same active perceptual categorization task (see also [15]).

### 5.3. Memory and Autonomy

The potential for structural diversity in the MBs allows to relate internal structure with function under different task conditions [30,68]. MBs that evolved to solve the “PathFollow” task with a need for associative memory (A2 and A4) developed more hidden units, and scored higher on many of the evaluated measures of autonomy and complexity (Figure 8).

As identified by $A_{4}^{S}$ (Figure 5), all agents adapted to task conditions A2 and A4 contained approximately 1 bit of “autonomous” information, compared to approximately 0 bit for task condition NA. However, given the particular task environments the agents were evolved to, this bit of information likely represents associative memory gathered from the environment and might converge to zero if more past sensor states are taken into account. Bertschinger et al. [1] based their autonomy measures on the notions of non-heteronomy (not being controlled by external factors) and self-determination. While the notion that an autonomous system should not be determined by the state history of the environment makes intuitive sense, for a large enough *m*, $A_{m}$ may ultimately only capture random noise intrinsic to the system’s units [1] (see also [2]). The need for memory and context-dependent behavior provides adaptive pressure for internal complexity and integration [30,91]. Memory does provide a system with autonomy from the immediacy of the environment. It may make sense to discount memory when evaluating particular aspects of autonomous behavior, but it should not be discounted altogether.

### 5.4. Correlation, Causation, and Internal Structure

Comparing the various measures related to autonomy on a data set of small artificial agents with diverse network architectures revealed important differences between approaches from different disciplines, particularly between information-theoretical and causal measures (even though some are based on the same formalism). For example, $A_{m}$ (evaluated as $A_{4}^{S}$, Equation (6)), consistently identifies the task-related need for memory of external inputs in conditions A2 and A4, but depends very little on an agent’s specific implementation (how the agent does what it does). By contrast, the causal version of the same measure, ${\hat{A}}_{m}$ (evaluated as ${\hat{A}}_{4}^{S}$, Equation (12)) varies considerably within task condition.

Similarly, there is no clear correlation between measures of informational closure ($J_{t}$ and $NTIC_{m}$) and $\langle \Phi^{\max}\rangle$. Information closure ($J_{t}$) evaluates the “information flow” from the environment into the system, while the integrated information ($\langle \Phi^{\max}\rangle$) of a system captures the irreducible causal constrains a system exerts onto itself, above a background of external influences.

In practice, causal measures are more difficult to evaluate, as they require knowledge about the causal interaction structure of the system [1], which corresponds to the system’s full TPM (Equation (1)). Since the MBs analyzed in this study are causal networks of interacting units, conceptually, there is little reason to choose information-theoretical over causal approaches when it comes to determining their degree of autonomy. As noted by Bertschinger et al. [1], purely observational measures may fail to disambiguate whether to attribute observed correlations to the system itself or the environment in the case of bidirectional interactions (see also [104]). By contrast, causal measures implement the idea that autonomy should be ascribed based on a system’s underlying mechanisms, as opposed to mere observation of the system’s behavior [1,105]. However, this implies that implementation—how a system does what it does—matters for autonomy. In other words, two systems that are equivalent in their behavior may still differ widely in their respective degree of autonomy.

As argued in [67,106], causal structure also matters for delineating the borders of a system from its environment, and for identifying whether the system under observation is in fact one system as opposed to multiple. While a strongly connected network architecture is necessary for Φ>0, the proposed measures of informational closure or dynamical complexity may yield similar results when applied to a “system” consisting of two or more independent modules as for a system that forms one unified whole.

### 5.5. Conclusions

Testing measures of autonomy and intelligence in artificial agents whose structure and function is known in all detail, forces ideas about autonomy to be made explicit and quantifiable. The measures reviewed in this study specifically capture three aspects of autonomy: a system’s self-determination, closure (independence from the environment), and agency. Comparing these measures on a data set of structurally diverse automata has moreover highlighted the role of implementation (how a system does what it does) for assessing whether and to what extent a system forms an autonomous agent. Finally, the “autonomy” toolbox accompanying this study makes all reviewed measures available for application to small, discrete dynamical systems, with the goal to focus future debates on intelligent behavior and its relation, or dissociation, to intrinsic intelligence, autonomy, and consciousness as integrated information [47,48,107]. While simple artificial agents like the ones employed in this study are only toy implementations of neural networks capable of truly complex behavior, they may still serve as a vehicle toward resolving theoretical disputes and clarifying conceptual confusions [3,31,108].

## Figures and Tables

**Table 1:** Agents were evolved under three task conditions. The table highlights the differences between conditions. All other parameters remained the same.

Condition	NA	A2	A4
Number of generations	50 k	150 k	150 k
Number of turn symbols	2	2	4
Random turn symbols	No	Yes	Yes
Number of evaluations per generation	1	10	10
Number of available sensors	4	4	5

**Figure A1:**
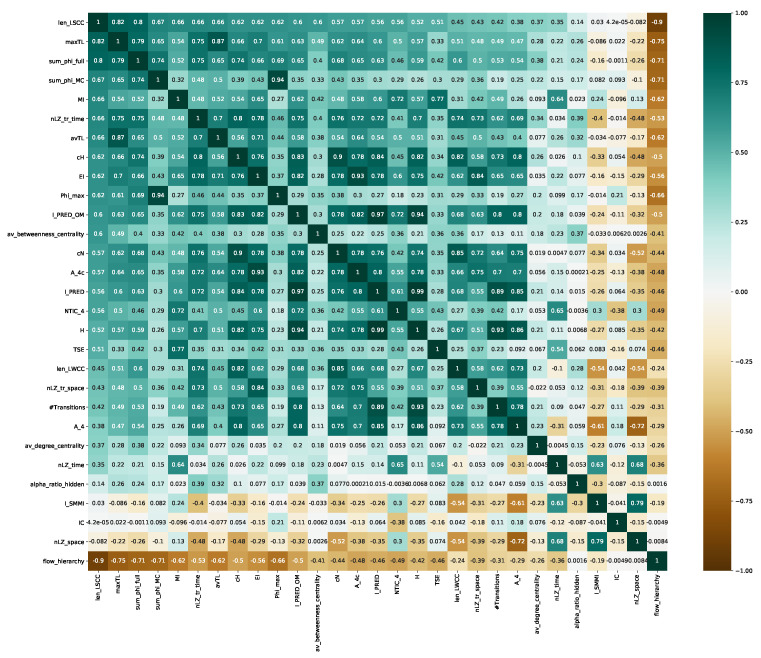
Correlation coefficients. Rows and columns are sorted by correlation with len_LSCC.

**Figure A2:**
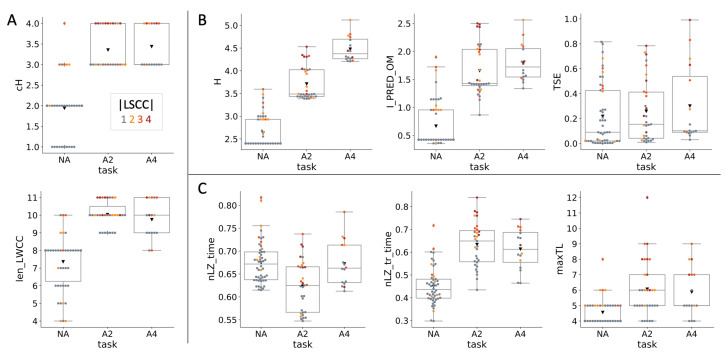
Additional evaluated quantities. (**A**) Structural measures: cH denotes the number of connected hidden units; len_LWCC the length of the largest weakly connected component. If len_LWCC is smaller than the number of connected units (Figure 4) the MB is constituted of two or more independent modules. (**B**) Information-theoretical measures: shown are the system entropy H (Equation (2)), $I_{pred}$ of the hidden and motor units, without the sensors, and $C_{TSE}$ (Equation (10)). (**C**) Dynamical measures: shown are the normalized Lempel-Ziv complexity (nLZ) reshaped along the time axis, applied to the MBs’ recorded activity (nLZ_time) and the MBs’ transients upon perturbation with fixed sensors (nLZ_tr_time), as well as the maximum transient lengths upon perturbation (maxTL).

## Data Availability

The data set evaluated in this study is available within the “autonomy” toolbox, which also includes the code to compute all evaluated measures at https://github.com/Albantakis/autonomy (accessed on 15 September 2021).
